# Skp2 expression is associated with high risk and elevated Ki67 expression in gastrointestinal stromal tumours

**DOI:** 10.1186/1471-2407-8-134

**Published:** 2008-05-13

**Authors:** Dolores Di Vizio, Francesca Demichelis, Sara Simonetti, Guido Pettinato, Luigi Terracciano, Luigi Tornillo, Michael R Freeman, Luigi Insabato

**Affiliations:** 1 Department of Functional and Biomorphological Science, University "Federico II", Naples, Italy; 2 Urological Diseases Research Center, Dept. of Urology, Children's Hospital, Dept. of Surgery, Harvard Medical School, Boston, MA, USA; 3 Department of Pathology, Brigham and Women's Hospital; Harvard Medical School, Boston, MA, USA; 4 Department of Pathology, University of Basel, Switzerland; 5 Dept. of Biological Chemistry and Molecular Pharmacology, Harvard Medical School, Boston, MA, USA

## Abstract

**Background:**

Gastrointestinal stromal tumors (GIST) exhibit an unpredictable clinical course and can rapidly progress to lethality. Predictions about the biological behavior of GIST are based on a number of canonical clinical and pathologic parameters whose validity in distinguishing between a benign and a malignant tumour is still imperfect. The aim of our study was to investigate the role of morphologic parameters and expression of cells cycle regulators as prognosticators in GIST.

**Methods:**

We performed an immunohistochemical analysis for Ki67, p27^Kip1^, Jab1, and Skp2, on a Tissue Microarray (TMA) containing 94 GIST. Expression of the above proteins was correlated to classically used prognosticators, as well as to risk groups. Clinical significance of histologic and immunohistochemical features were evaluated in 59 patients for whom follow-up information was available.

**Results:**

Overexpression of Ki67 and Skp2, and p27^Kip1 ^loss directly correlated with the high risk group (p = 0.03 for Ki67 and Skp2, p = 0.05 for p27^Kip1^). Jab1 expression did not exhibit correlation with risk. In 59 cases provided with clinical follow-up, high cellularity, presence of necrosis, and Ki67 overexpression were predictive of a reduced overall survival in a univariate model. The same parameters, as well as mitotic rate, tumour size, and p27^Kip1 ^loss were indicative of a shortened relapse free survival interval. High cellularity, and high mitotic rate retained their prognostic significance by multivariate analysis.

**Conclusion:**

Our data suggest that a number of histologic parameters in combination with immunohistochemical expression of cell cycle regulators can facilitate risk categorization and predict biologic behavior in GIST. Importantly this study demonstrates, for the first time, that Skp2 expression correlates with Ki67 expression and high risk in GIST.

## Background

GIST are the most frequent non-epithelial tumors of the GI tract. They originate from the interstitial cells of Cajal and are strongly dependent on signaling from the receptor tyrosine kinase (RTK) KIT and the KIT ligand, stem cell factor. Activating mutations in KIT and platelet derived growth factor receptor-A (PDGFRA) result in uncontrolled proliferation and ultimately trigger the onset of GIST [1,2]. Mutations in the *kit *gene in exons 11, 9, 13 and 17 represent early events in GIST onset and have been associated with malignancy [1,3]. Constitutive activation of RTKs induces expression and/or phosphorylation of a variety of intracellular proteins involved in cell survival, proliferation, and motility and adhesion signals, including components of the PTEN/PI3K/AKT pathway [4], which modulates cell cycle progression by down-regulation of p27^Kip1 ^in various neoplasms. Reduced expression of PTEN due to mutation is predictive of aggressive disease in human tumors [5]. Low or absent expression of PTEN in GISTs has been found to be associated with clinical progression and poor prognosis [6].

Tumour size and mitotic index play a key role in the grading system for GIST [1,2], although their value in predicting the biological behaviour is limited and all GIST, of any size, can potentially metastasize. To date no single morphologic, immuno-phenotypic or genetic marker can predict the aggressiveness of GIST [1].

The risk classification derived from a 2002 NIH consensus conference provided a means for hierarchical classification of aggressiveness into four categories: very low, low, intermediate and high risk, based on tumour size and mitotic rate [1]. This classification, while conferring essential information on prognostic criteria, still needs to be confirmed by using real follow-up data. In addition, the consensus criteria over-estimate the biologic potential of gastric GIST, in particular that of large tumors with low mitotic rate, as recently reviewed by Miettinen and Lasota [2]. However in general, gastric tumours have a more favourable prognosis than intestinal ones with similar parameters [7].

The proliferation marker Ki67 has proved useful in assessing the rate of tumour cell proliferation in GIST, although it does not seem to be more reliable than mitotic count [8]. Uncontrolled cell growth caused by a reduction of levels of cyclin-dependent kinase inhibitors (CKI) is a key event in human tumour evolution [9]. Loss of p27^Kip1 ^correlates with aggressive potential in human carcinomas [10], and sarcomas [11], and is an independent prognostic factor in other human tumours [12]. Published evidence suggests a more general deregulation of the cell cycle in GIST, and we have recently reported that p16^INK4A ^loss can identify high risk GIST [13], in agreement with data from a separate study [14]. p27^Kip1 ^loss has been associated with malignant potential in some reports on GIST [15-17], although its value as a prognostic marker has not been conclusively determined in this tumour type [18]. The human Jun activation-binding protein 1 (Jab1) has been identified as a p27^Kip1 ^interacting protein, that induces nuclear export of p27^Kip1 ^to the cytoplasm, thus permitting its degradation via the ubiquitin-proteosome pathway [19], and in particular by the activation of the SCF-Skp2/E3 ubiquitin protein ligase pathway [20]. The F-Box protein Skp2 functions as the substrate recognition factor of the SCF complex, which recognizes and binds to phosphorylated p27^Kip1 ^[21]. Recent findings indicate that p27^Kip1 ^contributes to Skp2 inhibition via a mechanism that involves repression of target gene promoter activity, suggesting a reciprocal regulation between the two proteins [22]. Skp2 overexpression plays a role as an independent prognosticator, stronger than p27^Kip1 ^and Ki67, in soft tissues sarcomas [23]. To the best of our knowledge, its significance as a prognostic marker in GIST has not been investigated.

The role of Jab1 in human oncogenesis is currently under investigation. Tomoda et al. showed that Jab1 physically interacted with p27^Kip1 ^and enhanced its cytoplasmic translocation, which resulted in accelerated degradation of p27^Kip1 ^via the ubiquitin/proteasome pathway [19]. Data on human tumours demonstrate that p27^Kip1 ^expression is inversely correlated to Jab1 expression [24], and recently Jab1 expression has been demonstrated to induce p27^Kip1 ^degradation by a Skp2 independent mechanism in pancreatic carcinoma cells [25].

In this study we have evaluated the significance of histologic parameters and expression of cell cycle regulators in 94 GISTs, 59 of which with clinical information. Our data confirm the importance of using a mixture of histologic and immunohistochemical parameters in order to predict potential benign or malignant behavior in a given GIST at diagnosis. They also point out to a role for Skp2 and p27^Kip1 ^as prognostic indicators, in GIST, of good or bad behavior respectively.

## Methods

### Clinico-pathologic evaluation

**Figure 1:**
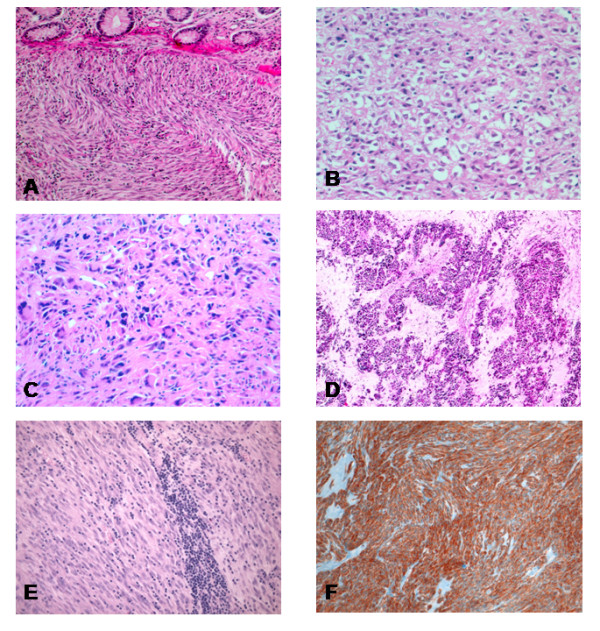
**Morphologic features of GISTs.** A) Spindle cell tumor infiltrating the submucosa and lamina propria of the colon. B) Epithelioid cell type tumor. C) Malignant GIST with prominent pleomorphism and high mitotic activity. D) Nuclear palisade feature of GISTs. E) Numerous scattered and patchy tumor infiltrating lymphocytes. F) Strong immunoexpression of CD 117.

Sample collection, tumour localization, assessment of behavior, and TMA construction have been described in detail elsewhere [3]. Two pathologists (DDV and LI) re-examined representative slides of each tumour. The diagnosis of GIST was confirmed in case of a mesenchymal spindle cell or epithelioid tumour of the GIST showing unequivocal positivity for CD117. 94 patients met these criteria and were included in this study. The 94 GIST patients comprised 50 males and 44 females with a mean age of 61 years (range 26–91, SD 14). Tumour localization involved stomach in 56 (56%), small bowel in 27 (27%), mesentery in seven (7%), duodenum in four (4%), rectum in three (3%), esophagus in one (1%), colon in one (1%), abdominal soft tissues in one (1%). The mean tumour size was 67.3 mm (range 5–250). A total of 37 tumours (37%) were < 50 mm in diameter. The assessment of behavior, based upon the categorization provided by the NIH consensus group in 2002, has been previously described [3]. Representative large sections of each tumour were evaluated for the following morphologic criteria: cell type, cellularity, mitotic rate, presence of tumour necrosis, and of giant cells [26]. In addition the presence of tumour infiltrating lymphocytes (TIL) was considered. Cell type was attributed determining the predominance (> 50%) of spindle, and epitheliod cells. Cellularity was scored, counting the number of cells in random areas > 0.1 mm^2 ^and considering a number of 100 cells per 10 high-power fields (HPF), as low cellularity (≤ 100 cells) and high cellularity (> 100 cells). Mitoses were evaluated with mitotic count per 50 high power fields (HPF), and the cutoff used was 5 mitosis per 50 HPF according to the 2002 NIH consensus conference [1]. Risk categories were established using the scheme provided by Fletcher et al [1], based on tumor size and mitotic count. The samples were consequently classified in group 0) very low risk tumours, group 1) low risk tumours; group 2) intermediate risk tumours, and group 3) high risk and malignant tumours, respectively. Tumor necrosis and giant cells were scored as 0–1 depending on their presence or absence. For TIL a 0–2 score depending on the abundance of lymphocytes infiltrating the neoplasms was used: 0) absence of lymphocytes; 1) presence of scattered lymphocytes; 2) grouped (patchy) lymphocytes (Fig. 1E).

### Immunohistochemical study

A panel of antibody probes, including antibodies against KIT (CD117), CD34, Smooth Muscle Actin (SMA), Desmin, S-100, Neuronal Specific Enolase (NSE), keratin, and Ki-67 (details in Table 1), were applied to whole sections of the tumours, using the ABC technique after antigen retrieval. The same markers were examined on the TMA and displayed good concordance with the whole sections (data not shown).

**Table 1:** Primary antibodies for immunohistochemical analysis

**Antibody**	**Clone**	**Manufacturer**	**Retrieval**	**Dilution**	**Method**
**CD117**	Polyclonal	DAKO, Carpinteria, CA, USA	Steam 120°/3' Cytrate buffer pH 6	1:300	ABC
**CD34**	QBEND10	DAKO, Carpinteria, CA, USA	Microwave 98°/30' Cytrate buffer pH 6	1:900	ABC
**CK22**	Cytokeratin	Biomeda, san Francisco, CA, USA	Microwave 98°/30' Cytrate buffer pH 6	1:400	ABC
**Desmin**	D33	DAKO, Carpinteria, CA, USA	Cook 15' Cytrate buffer pH 6	1:200	ABC
**SMA**	1A4	Sigma, Saint Louis, MO, USA	Microwave 80°/30' Cytrate buffer pH 6	1:60000	ABC
**S-100**	Polyclonal	DAKO, Carpinteria, CA, USA	Microwave 80°/30' Cytrate buffer pH 6	1:10000	ABC
**NSE**	BBS/NC/VI	DAKO, Carpinteria, CA, USA	Cook 15' Cytrate buffer pH 6	1:8000	ABC
**Ki67**	MIB-1	DAKO, Carpinteria, CA, USA	Microwave 98°/60' Cytrate buffer pH 6	1:16000	ABC
**p27**^Kip1^	57	BD Transduction Laboratories, Lexington KY, USA	Microwave 98°/60' Cytrate buffer pH 6	1:500	ABC
**Jab-1**	Polyclonal	Santa Cruz Biotechnology, Santa Cruz, CA, USA	Microwave 98°/60' Cytrate buffer pH 6	1:200	ABC
**Skp2**	2C8D9	Zymed LAB, San Francisco, CA, USA	Microwave 98°/60' Cytrate buffer pH 6	1:100	ABC

Immunohistochemical analysis of cell cycle regulators p27^kip1^, Skp2, Jab1 and Ki67 was performed using commercially available antibodies. The conventional avidin-biotin complex procedure was applied according to the manufacturer (DAKO, Carpinteria) protocol after optimization of antigen retrieval. For each antibody only neoplastic cells showing definitive nuclear staining were considered positive and semi-quantitative evaluation was performed. High nuclear expression of p27^kip1^, Skp2, and Ki67 was defined when > 50%, 10%, and 10% of nuclei were positive, respectively, according to Oliveira et al in soft tissue sarcomas [23]. For Jab1 we arbitrarily used 10% as a cutoff of positivity for the purpose of statistical analysis. The scoring was performed by a single pathologist (DDV), who had no prior knowledge of the clinical characteristics.

### Statistical analysis

Kaplan-Meier curves were used to visualize the histologic criteria and the protein expression with respect to Overall Survival (OS) and to Relapse Free Survival (RFS) data at the univariate level. Log-rank test was used to evaluate statistical significance of associations. Cox regression analysis was done for multivariate analysis with a stepwise approach (Wald statistics was applied for entering covariates). Chi-square test and Fisher exact test were used to evaluate correlations between variables. A p-value < 0.05 was considered significant in all the analysis.

Using the scheme provided by Fletcher et al [1], essentially based on tumour size and mitotic rate, the tumours were initially grouped into four categories. Given the limited number of samples provided with follow-up information, for both the univariate and multivariate analyses, risk groups were dichotomized to Group A and B, comprising A) very low and low risk, B) intermediate, high risk, and malignant tumours. Tumour size was dichotomized (group 1 ≤ 5 cm, group 2 > 5 cm).

### Ethical approval

Institutional Review Board approval was obtained before the initiation of this study.

## Results

### Pathological findings

Grossly the tumors were variable in shape. Some were well circumscribed, firm and whitish, with an irregularly whorled cut-surface. Others showed ill-defined outlines, soft or fleshy consistency, with necrotic or hemorrhagic areas. Microscopically the tumors were categorized as spindle or epithelioid depending on the most represented cell type (> 50%) throughout the tumor. Thus 44 tumors (47%) were predominantly composed of spindle cells (Fig 1A), 13 tumors (14%) of epithelioid cells (Fig 1B), and 37 tumors (39%) of both cell types, mixed in variable proportions. 30 cases of gastric tumors showed the characteristic perinuclear cytoplasmic vacuoles, whereas skenoid fibers were seen in 3 tumors located in the small bowel. Other peculiar histologic features are represented in Fig. 1C–E. The categorization of risk was as follows: follow-up was available in 59/94 cases. 23 of them were considered clinically malignant (12 died of disease and 11 were alive with metastatic/recurrent disease). Of the 36 cases with known follow-up remaining, 35 were alive with disease and 1 was dead for other causes; since the follow-up was not long enough we pooled them with the 35 cases for which clinical data were lacking. Of these 71 cases (36+35) 14 had metastasis at time of diagnosis or later and therefore were considered also malignant. Therefore 37 cases (23+14) were considered as "malignant". The remaining 57 cases were categorized according to the NIH consensus: 12 cases were considered high risk tumours, 19 tumours were intermediate risk, 21 tumours were low risk, and 5 tumours were categorized as very low risk. Surveys for the presence of tumour-infiltrating lymphocytes (TIL) revealed a low amount of TIL in 2 tumours, moderate amounts in 28 tumours and numerous TIL in 5 tumours. Of all histological parameters, mitosis (p = 0.001), necrosis (p = 0.02), and tumour size (p < 0.001) significantly correlated with risk groups dichotomized to two categories (A & B). The presence of giant cells did not exhibit any correlation with other parameters.

### Immunohistochemical findings

All tumours displayed strong immunoreactivity for CD117. The staining was localized in the cytoplasm, with either a granular or homogeneous quality (Fig 1F). Some differences of immunoreactivity were observed. In particular, in gastric tumours, CD34, Desmin and Smooth Muscle Actin (SMA) were positive in 98%, 9% and 41% of the samples respectively; while in extra-gastric tumours, they were positive in 65%, 0% and 56% of the samples.

### Cell cycle protein expression

**Figure 2:**
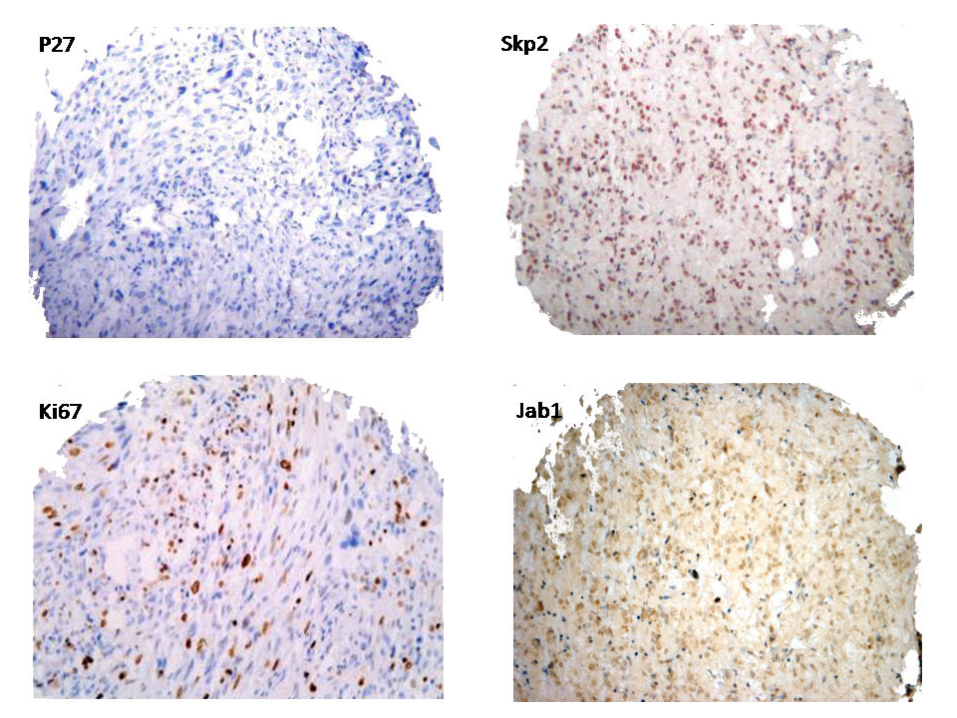
Composite figure of immunohistochemical staining showing p27Kip1 negativity and strongly positive Skp2, Ki67, and Jab1.

Detailed results are summarized in Table 2 and Table 3. Ki67 nuclear expression, higher than 10%, was identified in 21 tumours (19.7%), and directly correlated with high risk (p = 0.03). p27^Kip1 ^nuclear expression, in more than 50% of the cells, was detected in 16 tumours (15%) and was significantly higher in low-risk than in high-risk neoplasms (p = 0.04). Skp2 expression was detected in 24 tumours (32%), significantly higher in high risk tumours (p = 0.03). Representative examples of p27^Kip1^, Skp2, Jab1, and Ki67 staining are displayed in Fig 2. Jab1 expression was detected in 39 (36.6%) of the tumours and did not exhibit correlation with risk (data not shown). p27^Kip1 ^levels were directly and significantly correlated with Jab1 levels (p < 0.01), and an indirect trend of correlation was seen with Skp2 levels, even though this was not significant (data not shown). Interestingly Skp2 expression correlated with cellularity (p = 0.05), tumour size (p = 0.01), mitotic count (p = 0.05) and Ki67 (p = 0.01) (Table 3).

**Table 2:** Clinicopathologic parameters and risk categories

		Group A	Group B
N° cases		26	68

Sex	M	15	33
	F	11	35
Cell type	Spindle	8	36
	Epithelioid	4	9
	Mixed	14	23
Cellularity	Low	15	13
	High	11	55
Necrosis	0	26	48
	1	0	20
Mitosis	1	21	47
	2	5	21
Metastases		1	22
p27^Kip1 ^(%)		9/26 (34.6%)	7/50 (14%)
Skp2 (%)		4/25 (16%)	20/50 (40%)
Jab1 (%)		16/25 (64%)	23/48 (47.9%)
Ki67 (%)		3/25 (12%)	18/52 (34.6%)

Necrosis: 0 = absent; 1 = present

Mitosis: 1 ≤ 5 × 50 HPF; 2 > 5 × 50 HPF

Cellularity: Low cellularity ≤ 100 tumor cells × 10 HPF; High cellularity > 100 tumor cells × 10 HPF

**Table 3:** Correlation between morphologic criteria and cell cycle regulators.

**VARIABLE**	**p27**^Kip1^	**Ki67**	**Skp2**
**Cellularity**	1	< 0.001^a^	0.05^a^
**Mitotic count**	0.34	0.01^a^	0.05^a^
**Necrosis**	0.26	0.08	0.82
**Risk groups (A&B)**	0.04^a^	0.03^a^	0.03^a^
**Tumor size**	0.27	0.64	0.01^a^

^a ^Statistically Significant

### Clinico-pathological correlations and prognosis (Survival analysis)

**Figure 3:**
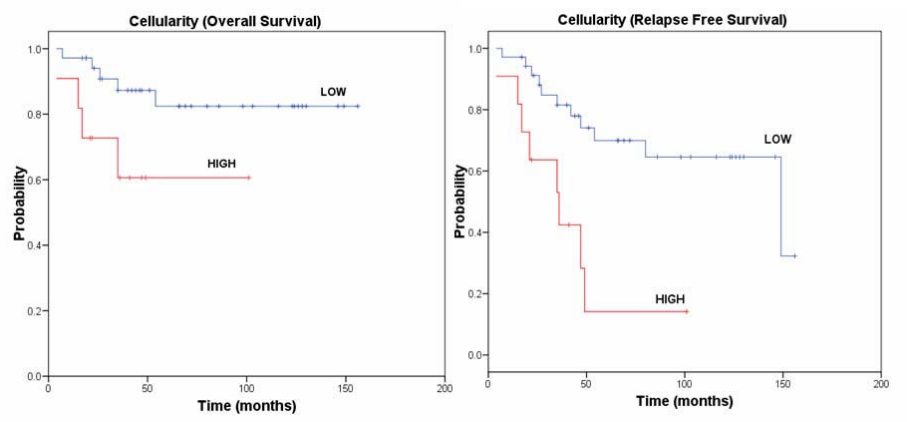
**Kaplan-Meier curves of cellularity with respect to overall survival and relapse free survival, respectively.** The p-value of the log-rank test is reported for each curve.

**Figure 4:**
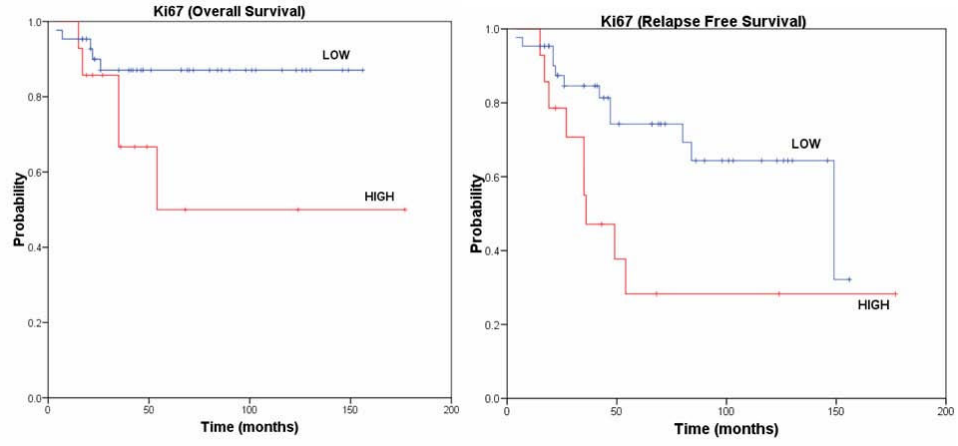
**Ki67 Kaplan-Meier analysis with respect to overall survival and relapse free survival, respectively.** The p-value of the log-rank test is reported for each curve.

The median follow-up period for the patients with GIST was 46 months (maximum 177, minimum 4). Medical charts were available in 59 cases of 94 patients (63%). Of these 59 patients, 11 (19%) died of disease, and 12 (21%) were alive with recurrent/metastatic disease. These 23 tumours were considered clinically malignant. An additional 35 patients (60%) were alive without disease, and 1 (2%) died from other causes. Univariate analysis was performed for patient age, tumour localization in the GI tract, tumour size, cellularity, cell type, mitosis, necrosis, risk grade, p27^Kip1^, Skp2, Jab1 and Ki67 expression. Of the above parameters, we found that necrosis (p = 0.01), high cellularity (p = 0.04) and Ki67 overexpression (p = 0.04) were significantly associated with reduced overall survival (OS). The best multivariate model included cellularity (p = 0.02) (data not shown). Cellularity (p = 0.01), mitosis (p < 0.001), tumour size (p = 0.01), high risk (p = 0.01), Ki67 (p = 0.02), and p27^Kip1 ^(p = 0.05) were significantly associated with reduced relapse free survival (RFS), by univariate analysis, as showed in Table 4. The Kaplan-Meier curves relative to cellularity and Ki67 expression are represented in Fig 3 and 4 respectively. TIL was associated with RFS, approaching statistical significance (data not shown).

**Table 4:** Univariate analysis of various parameters for Overall Survival (OS) and Release Free Survival (RFS).

**VARIABLE**	**OS**	**RFS**
**Cell Type**	0.42	0.27
**Cellularity**	0.04^a^	0.00^a^
**Mitotic count**	0.09	0.00^a^
**Necrosis**	0.01^a^	0.14
**Risk groups (A&B)**	0.07	0.01^a^
**Tumor size**	0.13	0.01^a^
**p27**^Kip1^	0.42	0.05
**Ki67**	0.04^a^	0.02^a^
**Skp2**	0.43	0.5

^a ^Statistically Significant

## Discussion

Our results, on a TMA containing 94 primaries GIST, show that morphological parameters and immunohistochemical markers, alone and in combination, may be useful in predicting benign or malignant biologic behavior of GIST at diagnosis. In particular, our analysis of 59 cases, for which clinical information was available, suggests 1) an independent role for high cellularity in predicting reduced OS; 2) a significant correlation of cellularity, mitosis, tumour size, high risk, Ki67 expression, and p27^Kip1 ^expression with RFS; 3) a direct correlation of p27^Kip1 ^loss as well as of Ki67 and Skp2 over expression with high risk; and 4) a significant correlation between Ki67 over expression and OS.

High cellularity, mitosis, and tumour size have been demonstrated to be prognostic factors in separate studies [2,8,27]. Our results confirm the validity of the above classical morphological parameters in attributing potentially malignant behaviour to a given tumour.

We also analyzed the tumours for the presence of TIL. We found a trend toward an inverse correlation between the presence of TIL and shortened RFS. TIL are the primary immune component infiltrating solid tumours and are considered to be a manifestation of the host anti-tumour reaction. To the best of our knowledge this feature, only analyzed in one recent report [28], has not been identified as a histologically significant indicator in GIST. Our data not only suggest a role for TIL in an anti-tumour reaction, but also suggest further investigation on additional series, into whether GIST patients might be identified who could benefit from immunotherapy.

In our study, Ki67 over-expression significantly correlated with high risk and with both OS and RFS. These data are in apparent contradiction with a study attributing a stronger potential to mitotic rate than Ki67 index as a prognosticator in GIST [15], however they are consistent with other studies in which Ki67 labeling index (LI) discriminated between potentially benign and malignant GIST better than counts of mitotic figures [8]. The direct correlation between expression of Ki67 and Skp2 was significant, in agreement with what has been found in several tumour types, including mesenchymal neoplasms [23,29,30].

In this study p27^Kip1 ^loss was significantly correlated with the degree of malignancy of GIST. The role of p27^Kip1 ^expression as a diagnostic or prognostic marker for a broad series of human tumours is currently under intense investigation. Our results are in agreement with a number of published studies [16,31,32]. In particular Gelen et al. demonstrated that p27^Kip1 ^expression is correlated to RFS [31]. p27^Kip1 ^loss has not been conclusively associated with biological aggressiveness of GIST, and is not used as a predictor of survival [15,17,18]. Our results, obtained on a larger series, show an association between p27^Kip1 ^loss and RFS, suggesting an important role for p27^Kip1 ^in the progression of GIST, and urging further investigation of its potential as a prognostic marker.

Cell cycle progression in mammalian cells is finely regulated by the sequential activation of a cascade of cyclin-dependent kinases, which are modulated by a complex series of interactions between cyclins and cyclin-dependent kinases inhibitors (CKIs). p27^Kip1 ^is a specific CKI present at high abundance in quiescent cells and is a putative tumour suppressor [11,33]. While loss of function of tumour suppressors occurs frequently at the genetic level, p27^Kip1 ^mutations represent an extremely rare event, consistent with data that p27^Kip1 ^expression is regulated at the post-transcripitonal level [34].

In our study, Skp2 expression significantly correlated with cellularity, high risk, tumour size, mitotic count, and Ki67 expression. To our knowledge, the significance of Skp2 over-expression as a prognostic marker in GIST has not been previously investigated. We found an inverse correlation between levels of Skp2 and p27^Kip1^, which approached the statistical significance. These data are in agreement with studies on lymphomas [35] and colorectal cancer [10] and suggest that over-expression of Skp2 contributes to p27^Kip1 ^degradation in GIST. Our data are in contrast with findings by Oliveira et al [23] in soft tissues sarcomas, which suggest the possibility of an alternative mechanism for p27^Kip1 ^degradation in sarcomas. Skp2 over-expression plays a role in aggressiveness of soft tissues sarcomas, where it has been found to be an independent prognosticator, stronger than p27^Kip1 ^and Ki67 [23]. In our study, Skp2 over-expression did not correlate with OS and RFS. However, the correlation of Skp2 expression with several parameters of malignant potential (cellularity, high risk, tumour size, mitotic count, and Ki67 expression) suggests an important auxiliary role of Skp2 in predicting the aggressive potential of GIST.

The role of Jab1 in human oncogenesis is currently under investigation. Recently Jab1 expression, inversely correlated to p27^Kip1 ^expression in pancreatic carcinoma and has been demonstrated to induce p27^Kip1 ^degradation by a Skp2-independent mechanism [25]. In our series, Jab1 expression did not correlate with any of the other parameters we analyzed, including p27^Kip1 ^expression, suggesting that degradation of p27^Kip1 ^in GIST might be Jab1-independent. It would be interesting to test this hypothesis using in vitro systems.

## Conclusion

We demonstrated in the current study that p27^Kip1 ^loss and Skp2 over-expression correlate with high risk grade in GIST. Additional studies are necessary to clarify their role as prognostic indicators.

## Competing interests

The study was funded by Novartis-Stiftung für medizinisch-biologische Forschung.

The authors declare that there are no other competing interests

## Authors' contributions

DDV designed the study, performed the immunohistochemical assay, analyzed the histologic and immunohistochemical results, and helped to draft the manuscript. FD performed the statistical analysis, and along with SS, helped to draft the manuscript. LTe, GP, LTo and MRF participated in the design of the study and MRF edited the manuscript. LI conceived the study, analyzed the histologic and immunohistochemical results, and helped to draft the manuscript. All authors read and approved the final manuscript.

## Pre-publication history

The pre-publication history for this paper can be accessed here:


